# Fruit Waste Potential for Single Cell Protein Production in Addis Ababa City, Ethiopia: A Review

**DOI:** 10.1002/fsn3.71177

**Published:** 2025-11-09

**Authors:** Gebeyehu Ayele, Habtamu Admassu, Gadissa Mosisa, Abebe Desalegn, Molla Abeje

**Affiliations:** ^1^ Department of Food Engineering, College of Engineering and Technology Dilla University Dilla Ethiopia; ^2^ Food Process Engineering Program, Department of Chemical Engineering, College of Engineering Addis Ababa Science and Technology University Addis Ababa Ethiopia; ^3^ Biotechnology and Bioprocess Center of Excellence Addis Ababa Science and Technology University Addis Ababa Ethiopia; ^4^ Department of Food Engineering, Faculty of Engineering Wollega University Nekemit Ethiopia; ^5^ Department of Food Engineering, College of Engineering and Technology Wolkite University Wolkite Ethiopia

**Keywords:** circular bio‐economy, fruit waste, microbial fermentation, protein scarcity, SCP

## Abstract

Urban food waste presents a significant environmental challenge, but it also offers a promising opportunity for sustainable protein production. In Addis Ababa, it's estimated that around 70%–74% of municipal solid waste is biodegradable, with a large portion made up of fruit and vegetable residues; bananas, mangoes, avocados, and citrus fruits dominate these organic streams. These high‐moisture, nutrient‐rich materials are particularly well‐suited for single‐cell protein (SCP) production through microbial fermentation, positioning them as a climate‐resilient alternative to conventional animal or plant‐based proteins. Current research explores various aspects: substrate composition, effective pretreatment strategies, selecting optimal microbial strains, different fermentation approaches, and crucial safety issues such as nucleic acid content, allergenicity, and potential mycotoxin contamination. Globally, SCP yields range from 20 to 50 g/L, but translating these results to Addis Ababa is complicated by local variability in feedstock quality and seasonal availability. This means solutions must be tailored to the specific context. Scaling up SCP production isn't without hurdles. Major challenges include the heterogeneous nature of the feedstock, its high moisture content, limited cold‐chain infrastructure, and noticeable gaps in technical expertise. Despite these challenges, there are tangible avenues for progress: establishing pilot‐scale fermentation facilities, developing decentralized preprocessing and collection systems, and embedding these efforts within broader circular bio‐economy frameworks. Together, these strategies could enhance local protein availability, reduce landfill contributions, and help curb greenhouse gas emissions. Still, several knowledge gaps remain particularly regarding Ethiopia‐specific techno‐economic analyses, process optimization for mixed fruit waste, and capacity‐building in fermentation biotechnology. Addressing these gaps is essential for moving SCP production from the laboratory to a viable, city‐wide solution. By integrating technological, regulatory, and industrial strategies, Addis Ababa can leverage fruit‐waste valorization to address urban organic waste, strengthen nutritional security, and further Ethiopia's goals in advancing a circular bio‐economy.

## Graphical abstract

Valorization of urban fruit waste for SCP production.

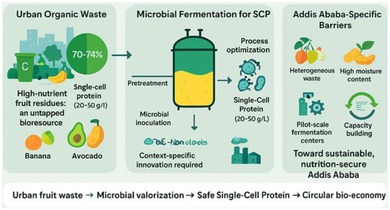

## 1 Introduction

Food waste has significant consequences for resource management, environmental quality, and food security, thus being one of the 21st century's most urgent concerns regarding sustainability. Globally, about 1.3 billion tonnes of food are lost or wasted every year in the supply chain, or about one‐third of all food produced for human consumption (FAO G 2011; FAO 2013). According to more recent estimates, between harvest and retail, 13% of food is lost, and food waste at the consumer, retail, and service sectors alone accounts for over 931 million tonnes annually (Forbes 2021). In addition to being a missed chance to reduce hunger, this inefficiency is a major cause of environmental damage because lost food reflects the work, energy, water, and land that went into its creation. According to environmental experts, food loss and waste (FLW) accounts for 8% to 10% of all greenhouse gas (GHG) emissions worldwide, making it one of the major emission sectors when viewed as a nation (Crippa et al. 2021; Rosenzweig et al. 2020). The effects are not limited to climate change: food that is never eaten accounts for roughly 30% of agricultural land and 25% of freshwater withdrawals worldwide (Kummu et al. 2012). According to the United Nations Environment Programme (UNEP) Food Waste Index Report 2024 (UNEP 2024), approximately 1.05 billion tonnes of food were wasted globally in 2022, accounting for about 19% of the food available to consumers at the retail, food service, and household levels. This staggering figure underscores the urgent need for sustainable solutions to address food waste and its associated environmental impacts. In areas that are already under water stress, this waste increases strain on water supplies while also hastening biodiversity loss, land degradation, and deforestation.

Global population growth, urbanization, and shifting dietary requirements are all contributing to the significant increase in demand for high‐quality protein. According to Sobhi et al. (2023), the traditional methods of producing meat, dairy, and fish are linked to significant greenhouse gas emissions and other environmental effects, as well as high demands on land, water, and energy. The protein known as Single Cell Protein (SCP), has shown promise as an enhancement or substitute for traditional protein sources. SCP provides an affordable, manageable, potentially more sustainable method of producing tremendous quantities of nutritious protein from a variety of feedstocks, including organic waste that would otherwise be thrown away (Muazzam et al. 2025; Sekoai et al. 2024). SCP production from fruit‐processing wastes and municipal organic waste deserves serious attention as part of an integrated circular‐bio economy approach in a context like Addis Ababa, Ethiopia, where urgent urban food demand and food loss in the supply chain coexist.

Ethiopia is currently home to more than 135 million people, most of whom are young. Rapid urbanization and population increase are putting more strain on the country's protein supply, especially for low‐income households in cities (Sida et al. 2025). While dietary studies show low median protein intakes (≈41 g/day) and limited consumption of animal‐source protein among women of reproductive age, national nutrition surveys continue to report high burdens of under‐nutrition, with stunting common among children (Woldeyohannes et al. 2023; Roba et al. 2023). Concurrently, Addis Ababa generates a substantial volume of biodegradable municipal waste products, of which market leftovers of fruits and vegetables make up a sizable portion of an underutilized, fermentable resource (Gelan 2021; Beka and Meng 2021). As an alternative to climate‐sensitive agriculture, turning this fruit waste into single‐cell protein (SCP) through the use of yeasts, fungi, or bacteria offers an affordable, water‐ and land‐efficient method of producing high‐quality protein and essential amino acids (Onyeaka et al. 2022). Under resource restrictions and continual drought, recent evaluations of Ethiopia highlight the possibility of SCP production as a climate‐resilient strategy to improve food security (Buchner et al. 2024). In Ethiopia, turning market fruit waste into SCP could both alleviate the environmental burden of disposing of organic waste and offer reasonably priced safe protein ingredients for food fortification or blended flours, thereby addressing protein scarcity and promoting circular bio‐economy pathways.

Several research gaps that impede the successful assessment of fruit waste as a substrate for single‐cell protein (SCP) production in Addis Ababa are revealed by a careful review of the literature. First, although research has shown that *Saccharomyces cerevisiae* can convert fruit peel residues, such as Beles (cactus pear), into SCP, these studies frequently optimize protein yield using inorganic nitrogen or added glucose, which may not accurately reflect the substrate conditions or resource limitations in the urban environment of Addis Ababa (Haddish 2015). Second, the majority of SCP research only looks at initial biomass yields and process optimization, ignoring downstream assessments of product safety and nutritional viability, such as digestibility, nucleic acid content, and potential pathogens—all important considerations for food and feed applications (Zhuang et al. 2024; Salazar‐López et al. 2022). Third, despite the abundance of fruit and vegetable waste streams in the area, there are tropical fruits such as banana, avocado, mango, and papaya in Addis Ababa; nevertheless, very little is known about the compositional and antinutritional profiling of this area (Yohannes et al. 2024; Gebreeyessus and Demessie 2014). Lastly, present research mostly focuses on laboratory‐scale viability, glaringly ignoring socio‐economic factors such as scalability, regulatory frameworks, and consumer acceptance in the Ethiopian setting (Salazar‐López et al. 2022; Sekoai et al. 2024).

## 2 Materials and Methods

This review was carried out by conducting a systematic search and analysis of peer‐reviewed publications, policy documents, and literature pertaining to the synthesis of Single Cell Protein (SCP), microbial fermentation, and fruit waste management. Relevant publications were found using keyword combinations such as “fruit waste,” “single cell protein (SCP),” “microbial fermentation,” “protein alternatives,” “food waste valorization,” “Ethiopia,” and “Addis Ababa” in major scientific databases such as PubMed, Scopus, Web of Science, ScienceDirect, and Google Scholar. To achieve thorough coverage and refine the search, boolean operators (AND, OR) were used. To enhance transparency and reproducibility, the review applied explicit inclusion criteria: studies published between 2010 and 2025 were prioritized to capture recent advances in SCP production and fruit waste valorization, while older foundational works were included selectively for historical context.

Either experimental or review publications that discussed the effects of fruit waste on the environment, the nutritional composition of fruit residues, the use of microbes to produce SCP, or case studies pertinent to Ethiopia and Sub‐Saharan Africa were taken into consideration. Books that were unavailable in English, didn't have the entire content, or weren't related to SCP manufacturing were not included. Fruit waste types and volumes, microbial strains, fermentation techniques, nutritional profiles, and the financial or environmental effects of SCP production were among the data taken from the chosen studies. Prioritizing peer‐reviewed publications, FAO/UN reports, and respectable academic institutions helped to assure the reliability of the sources. Findings from many studies were combined and contrasted to determine current patterns, identify research needs, and prospects for producing SCP from fruit waste in the Addis Ababa context.

## 3 The Concept of Single Cell Protein (SCP)

### 3.1 Definition and Significance of SCP

The term “single‐cell protein” describes protein‐rich microbial biomass, usually yeasts, filamentous fungi, bacteria, or microalgae that are grown for food or feed frequently on low‐value “second‐generation” substrates like fruit and food processing wastes, which are common in cities like Addis Ababa (Salazar‐López et al. 2022). Compared to conventional protein sources, SCP has the following advantages: very high protein yields (typically between 30% and 80% of dry weight) with advantageous essential amino acid profiles (particularly lysine, which enhances diets based on cereals); quick, year‐round production that doesn't require arable land; and significantly reduced land, water, and greenhouse gas footprints when incorporated into circular systems that value organic waste (Ye et al. 2024). In comparison to animal and many plant proteins, recent reviews and techno‐economic/LCA assessments show that using food and fruit wastes as feedstocks can lower costs while closing nutrient loops and reducing emissions. This makes SCP a scalable, urban‐adjacent option for reasonably priced nutrition and feed (Sekoai et al. 2024). These revisions further enhance SCP's cost competitiveness and sustainability by synthesizing developments in microbial selection and process engineering that boost output and safety (Li et al. 2024).

### 3.2 SCP Production Methods

**FIGURE 1:**
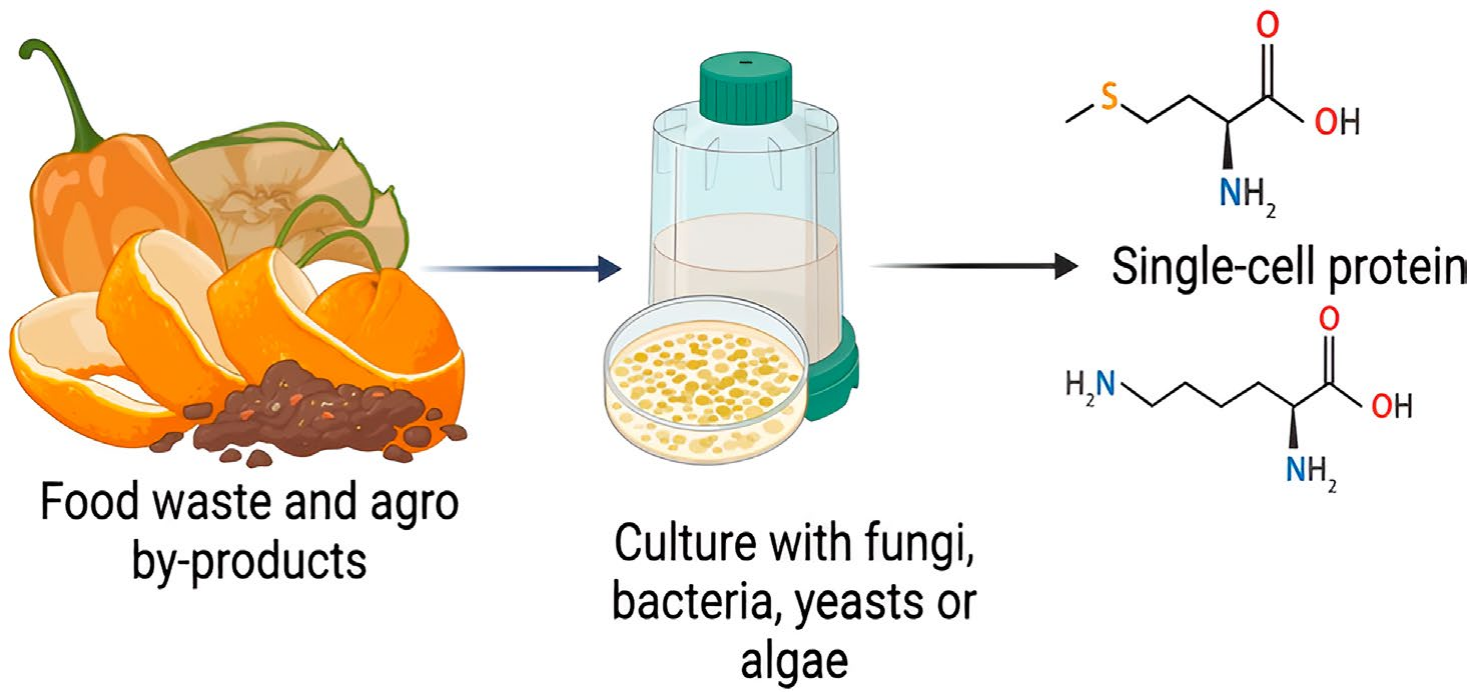
Single‐cell protein from food waste and agro by‐products.

Low‐cost lignocellulosic and carbohydrate‐rich residues, particularly agricultural by‐products and fruit and vegetable wastes, are significant in the SCP literature as the most scalable non‐grain feedstocks due to their abundance, year‐round availability, and suitability for straightforward physicochemical or enzymatic pretreatments that liberate fermentable sugars for bacteria, algae, filamentous fungi, and yeasts (Figure 1). According to recent reviews, crop residues (such as straw, husks, and bagasse), processing side‐streams, and mixed postharvest rejects can produce high cell densities while cutting the link between freshwater demand and protein production and arable land, which is crucial for areas experiencing food insecurity (Gao et al. 2025; Koukoumaki et al. 2024). In the fruit and vegetable category, following mild acid/alkali, steam, or enzymatic pretreatment, peel and pulp wastes from citrus, banana, mango, papaya, tomato, and mixed market discards have consistently supported robust SCP fermentations. Among the most successful producers are yeasts like *Saccharomyces, Candida*, and *Yarrowia* (Thiviya et al. 2022). Particularly in Addis Ababa, organics make up the majority of municipal solid waste streams; roughly 70%–74% of household waste is biodegradable, and the city's abundant fruit and vegetable markets produce large, concentrated feedstock nodes, suggesting a strong local potential to valorize these wastes into SCP while reducing landfill pressure (Gelan 2021; Teshome 2021). In terms of methodology, current approaches combine strain selection or metabolic engineering with substrate fractionation and detoxification to enhance nitrogen assimilation, amino acid profiles, and product safety. This approach is in line with Ethiopia's circular bio‐economy objectives (Li et al. 2024; Zhuang et al. 2024).

The diverse metabolic processes known as microbial fermentation, which are carried out by yeasts, bacteria, and filamentous fungi, are the foundation of both conventional food fermentations and contemporary biotechnological platforms for the production of single‐cell protein (SCP). Under controlled conditions, microorganisms transform carbohydrates and other organic substrates into cellular biomass and value‐added metabolites (Praveen and Brogi 2025). While bacteria (lactic acid bacteria, Bacillus, and mixed microbial consortia) offer fast growth on a variety of substrates and can improve substrate digestibility through extracellular enzymes, yeasts like *Saccharomyces* spp. and *oleaginous* yeasts flourish in sugar‐rich, frequently anaerobic or oxygen‐limited environments and are valued for their rapid biomass accumulation and favorable amino‐acid profiles (Zhuang et al. 2024; Kumar, Dhiman, et al. 2024). According to Khan et al. (2024) and Gebre et al. (2023), filamentous fungi, such as *Aspergillus* and *Rhizopus*, are excellent at solid‐state fermentations and secrete strong hydrolytic enzymes that transform complex fruit wastes, such as pectins and cellulose, into fermentable sugars. This allows for the effective conversion of agro‐food residues into microbial protein. The physiology of microorganisms (aerobic versus heterotrophic growth, enzyme secretion, and nucleic acid content) must be matched with the composition of the feedstock and the process mode (submerged, solid‐state, or fed‐batch) to maximize SCP yield, nutritional quality, and economic viability for localized applications like fruit‐ waste valorization in Addis Ababa, according to recent reviews (Li et al. 2024; Sekoai et al. 2024).

While *Saccharomyces cerevisiae* remains a staple in microbial protein production, largely due to its rapid growth and advantageous amino acid profile, it is evident that significant trade‐offs exist among different microbial groups. For example, *Kluyveromyces marxianus* demonstrates superior biomass productivity and thermal tolerance relative to *S. cerevisiae* ; however, it also necessitates more precise oxygen management to avoid unwanted ethanol production (Koukoumaki et al. 2024). Filamentous fungi such as *Aspergillus* are notably effective at hydrolyzing complex polysaccharides present in fruit peels, yet their tendency to accumulate excessive nucleic acids may restrict their direct application as animal feed unless additional processing steps are undertaken (Khan et al. 2024). These differences underscore the critical importance of matching specific substrates to appropriate microbial strains, rather than presuming uniformity in outcomes across taxa. Furthermore, discrepancies reported in the literature frequently stem from variations in pretreatment protocols: enzymatic hydrolysis typically results in enhanced fermentable sugar yields with minimal inhibitory byproducts, whereas acid hydrolysis often leads to the formation of furfural and HMF, both of which can inhibit microbial growth unless detoxification is implemented (Praveen and Brogi 2025). In the context of Ethiopia, the scalability of these biotechnological approaches must be critically assessed against existing infrastructure limitations. For instance, Addis Ababa generates approximately 0.45–0.5 kg of waste per capita daily, with over 70% consisting of biodegradable organics (CSA 2023; Gelan 2021). Nevertheless, centralized waste segregation is still lacking, which poses significant challenges to achieving consistent feedstock quality. Consequently, establishing decentralized preprocessing facilities near high‐volume markets such as Atikilt Tera, which processes several hundred tons of fruits and vegetables each week may offer a more practical pathway for single‐cell protein (SCP) feedstock collection and pretreatment. Ultimately, these considerations highlight the necessity of adapting global SCP technologies to local conditions, rather than assuming their direct transferability.

### 3.3 SCP As an Alternative Protein Source in World and Africa

Single‐cell protein production has recently emerged as a significant and practical avenue for addressing contemporary challenges relating to food security, environmental degradation, and the broader ambitions of a circular bio‐economy. This research examines consumer attitudes toward SCP in Addis Ababa, Ethiopia, situating these insights within a broader, rapidly evolving global context. Globally, SCP has moved well beyond its initial pilot stages. In regions such as Europe and North America, companies like Quorn, which utilizes the filamentous fungus *Fusarium venenatum*, and Nature's Fynd, which leverages microbes sourced from geothermal springs, have successfully launched SCP‐based products into mainstream retail markets. This not only demonstrates commercial feasibility but also signals an increasing level of consumer acceptance (Finnigan et al. 2019; Linder 2019). Complementing these commercial efforts, the European Union's “ProFuture” initiative is actively investing in scaling up microalgal and fungal protein production, further signaling SCP's growing strategic importance in developed economies (ProFuture 2022). The motivations for SCP adoption differ across regions. In industrialized nations, the primary focus tends to be on minimizing the ecological impact of traditional meat production, as well as developing innovative, health‐oriented food products (Järviö et al. 2021). By contrast, in parts of rapidly industrializing Asia, SCP production is frequently coupled with waste valorization. For example, ongoing research in Southeast Asia is exploring the conversion of palm oil mill effluent into microbial biomass for animal feed, thereby addressing both environmental and nutritional concerns (Onyeaka et al. 2022). Overall, these developments collectively indicate that SCP is no longer a theoretical or futuristic concept. Rather, it represents a tangible and versatile solution, with applications and adoption strategies tailored to the specific resources and priorities of each region.

Through the conversion of plentiful agro‐food residues into microbial biomass rich in essential amino acids and micronutrients, single‐cell protein (SCP) production from fruit waste provides a practical, immediately deployable method to increase affordable, high‐quality protein in Ethiopia and similar regions (Thiviya et al. 2022; Li et al. 2024). Small‐scale bioreactors, low‐cost local substrates (peels, pulp, and bagasse), and the location of SCP processes near fruit‐processing hubs (like Addis Ababa) can all help communities reduce waste and improve nutrition while lowering supply‐chain and transportation costs and aligning with circular economy goals (Buchner et al. 2024; Thiviya et al. 2022). The generated biomass can be used as a high‐value feed ingredient to increase local animal production or as a direct protein supplement in fortified foods, increasing its impact on dietary diversity and household food security (Chamodi et al. 2025; Onyeaka et al. 2022). A robust and scalable protein source could be provided by incorporating SCP into national nutrition strategies, especially when paired with strict processing and safety controls (to eliminate excess nucleic acids and contaminants), and community‐level education to ensure acceptability, given Ethiopia's high rate of undernutrition and recurrent acute food insecurity (Amaha 2020; Zhuang et al. 2024). Therefore, before widespread adoption, more research, pilot projects, and funding for regional value chains are required to confirm cost‐effectiveness, legal routes, and cultural acceptance (Koukoumaki et al. 2024; Li et al. 2024).

## 4 Fruit Waste as a Potential Substrate for SCP Production

### 4.1 Types of Fruit Waste in Addis Ababa

**FIGURE 2:**
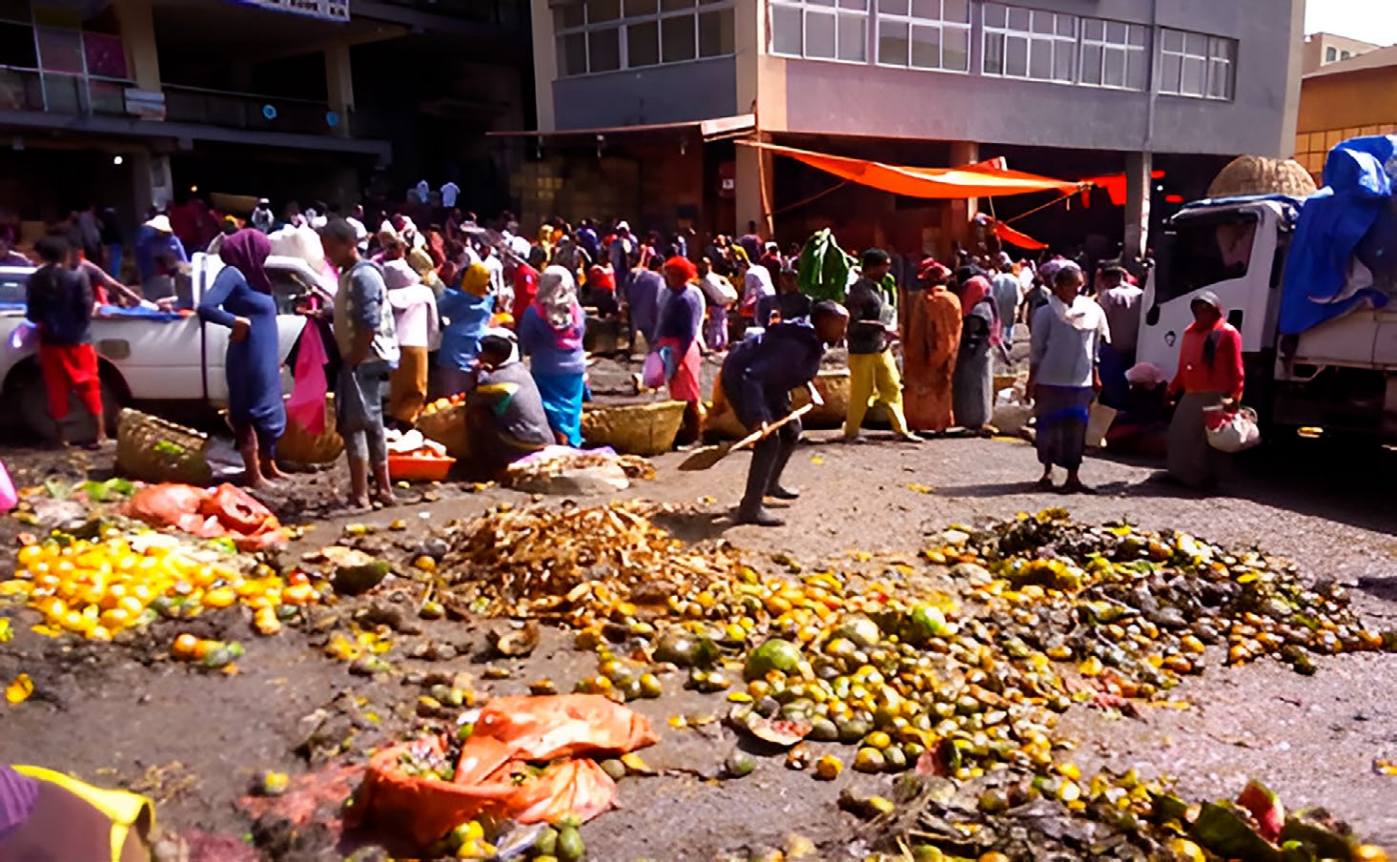
The fruit waste from Addis Ababa, “atiklit tera” market place.

Whole overripe or bruised fruits and processing residues from small‐scale and household vendors, primarily bananas, mangoes, avocados, and citrus fruits, account for the majority of fruit discards in Addis Ababa (Figure 2). Moreover, a large amount of peels, seeds, pulp (pomace), and crushed flesh (overripe pulp) enter the municipal organic stream (Ababa 2018). Together, these wastes account for a significant portion of the city's biodegradable fraction (high organic content recorded in current municipal waste characterizations), which is often generated at marketplaces, street vendor stalls, and from domestic trimming or juice/food preparation. Fruit peels (bananas, citrus, etc.) are common and volumetric, while pulp and pomace accumulate where fruit processing and informal juice take place. These fractions are rich in micronutrients, fiber, and fermentable sugars, which make them desirable substrates for microbial valorization processes like the production of single‐cell proteins (Duguma et al. 2024). The primary discard categories are regularly identified with research on Ethiopian urban fruit waste streams and studies of fruit by‐products conducted worldwide emphasizing their suitability for bioconversion (Mersha and Manczarski 2025).

**FIGURE 3:**
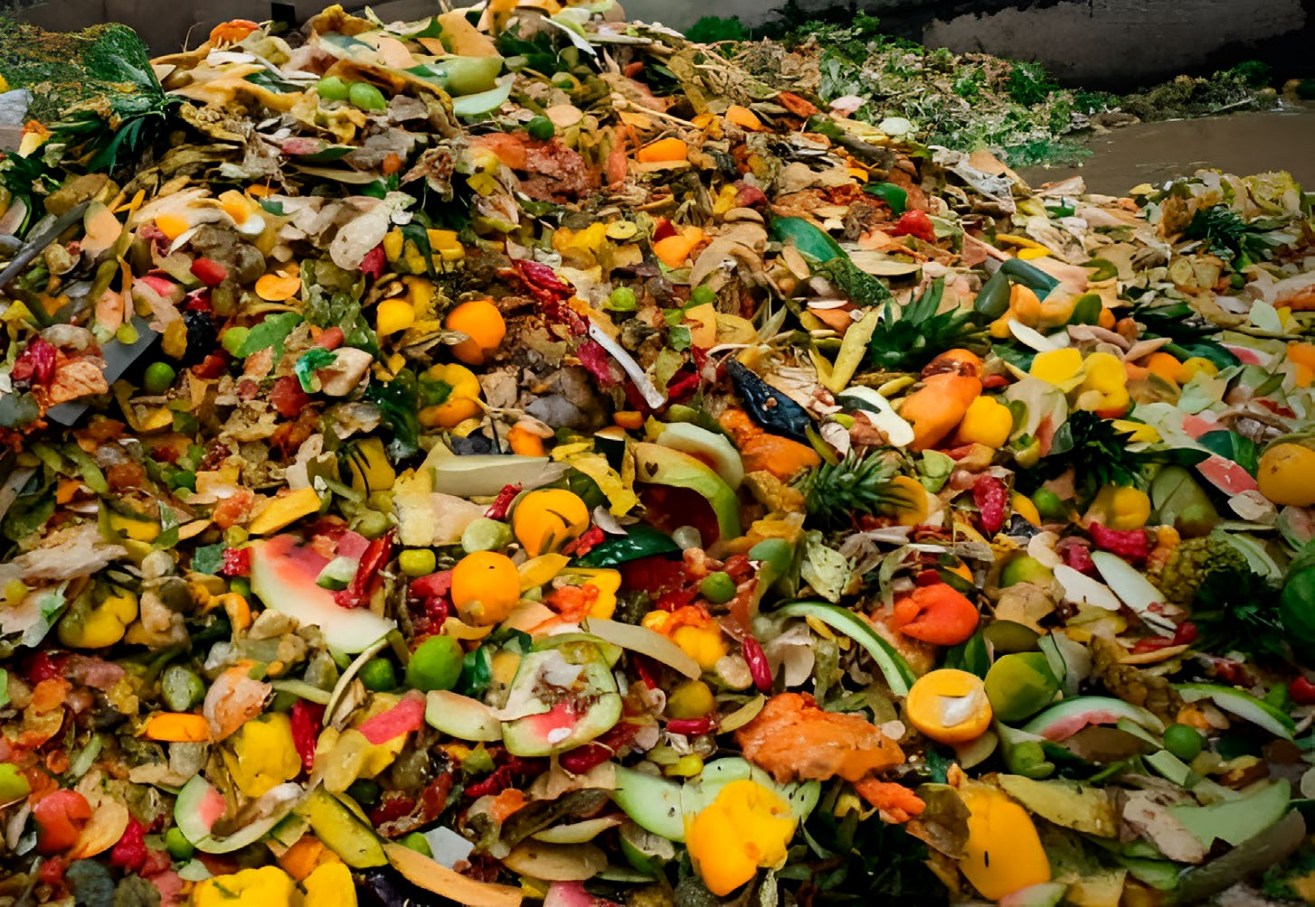
Abundant fruit waste streams in Addis Ababa, Ethiopia as a potential resource for single cell protein production.

The high proportion of food‐derived municipal waste in Addis Ababa and the pronounced seasonal production cycles for key fruits (banana, mango, and avocado, citrus) also contribute to the large quantities and seasonal variations in fruit‐waste generation in Addis Ababa (Figure 3). Ethiopian municipal solid‐waste production averages around 0.38 kg/person‐day, with organic (food and agricultural) fractions occupying urban waste streams often reported between roughly 50%–70% of total waste so fruit residues are a significant share of the organic fraction in Addis Ababa's mix (Gebrekidan et al. 2024; Kassahun et al. 2025). Large, season‐locked losses that increase available biomass during harvest months are documented by studies of mango value chains and post‐harvest loss estimates. National production trends (fruit output has increased significantly over the past ten years) and concentrated harvest windows for citrus, bananas, and mangoes produce distinct seasonal peaks in market and post‐harvest losses, which in turn produce temporal surges of peel, pulp, and cull material entering urban collection systems (Adame et al. 2023; Tarekegn and Kelem 2022). Last but not least, research on local waste quantification and forecasting for Ethiopian cities reveals both an increase in the overall amount of organic waste as well as quantifiable seasonal variability findings that suggest the availability of single‐cell protein feedstock in Addis Ababa will vary predictably with harvest calendars and market cycles, requiring seasonally adaptive collection, storage (e.g., processing techniques such as ensiling and drying) for reliable SCP production (Teshome et al. 2023; Obsa et al. 2022).

Recent studies have drawn attention to the considerable scale and concentration of fruit waste in Addis Ababa's major marketplaces, particularly Atikilt Tera, Shola, and several regional collection hubs. For instance, Kassahun et al. (2025) document that Atikilt Tera alone generates several hundred tons of organic residues per week, with fruit‐based materials accounting for approximately 30%–40% of that total. The aggregation of such substantial volumes of organic matter in centralized market locations is highly relevant for single‐cell protein (SCP) valorization, as it facilitates a continuous and readily accessible supply of peels, pulp, and spoiled fruit materials that would otherwise be discarded in unmanaged dumping sites. This clustering effect not only lowers collection costs but also makes it feasible to situate pilot‐scale microbial conversion units near existing waste aggregation points, which is particularly significant given Addis Ababa's limited formal infrastructure for waste segregation. Another notable factor specific to Ethiopia is the unique composition of its fruit residues. Analytical data on discarded banana and mango fractions from Addis Ababa markets reveal higher moisture and soluble sugar contents than those typically found in the drier peels of temperate regions (Gebrekidan et al. 2024). While this compositional profile enhances the fermentability of the residues for yeasts and lactic acid bacteria, it simultaneously increases the risk of rapid microbial spoilage if wastes are not quickly processed. Therefore, preservation strategies such as ensiling, solar drying, or other low‐cost stabilization methods are particularly important for Ethiopia's warm climate, where ambient temperatures may exceed 25°C–28°C for much of the year. The integration of such preservation steps has the potential to mitigate seasonal losses and ensure a consistent substrate supply for SCP production. Moreover, Ethiopia's rapidly expanding urban population and evolving dietary patterns are reshaping the landscape of fruit waste generation. National data (CSA 2023) indicate that urban fruit consumption has grown by more than 30% over the past decade, with imports of bananas and citrus fruits supplementing domestic production during off‐seasons. This shift results in both larger and more continuous flows of peel and pulp residues compared to the more seasonal patterns observed in rural settings. These dynamics suggest that Addis Ababa is particularly well‐suited to serve as a testing ground for circular bio‐economy models that transform otherwise unmanaged fruit waste into microbial protein for food and feed applications. Nevertheless, for such waste‐to‐protein initiatives to succeed, they must be carefully aligned with the practical realities of existing infrastructure. This includes accommodating informal collection networks and navigating limited cold‐chain capacity to design SCP valorization systems that are both pragmatic and tailored to the city's current context.

Although this review primarily addresses the valorization of fruit waste for single cell protein (SCP) production in Addis Ababa, Ethiopia, it is essential to recognize the broader relevance of these findings. Globally, fruit and kitchen waste are increasingly valued as substrates for bio‐based industries, owing to their abundance, nutrient content, and biodegradability. For instance, fruit waste has long been utilized in bioethanol production due to its carbohydrate‐rich and lignocellulosic composition, which facilitates efficient fermentation (Mgeni et al. 2024). Beyond biofuel applications, kitchen and fruit wastes are also being harnessed for microbial enzyme production, providing a cost‐effective alternative to traditional synthetic media (Poddar et al. 2023). Recent research further underscores the potential of fruit waste‐derived macromolecules for the development of value‐added biomaterials. Extracted polysaccharides, proteins, and phenolic compounds from fruit by‐products are being repurposed as precursors for bioplastics, resins, and nano‐materials, thereby highlighting their multifunctional utility (Vieira et al. 2025). The utilization of kitchen wastes for bioplastic production, in particular, represents an environmentally conscious strategy that addresses plastic pollution and advances circular economy models. In the enzyme industry, citrus by‐products have emerged as especially promising, offering cost‐effective feedstocks for the fermentation of industrial enzymes such as cellulases, pectinases, and amylases enzymes that are crucial across food, feed, and bioprocessing sectors (Lima et al. 2025). Collectively, these international advancements illustrate that the valorization of fruit and kitchen wastes is not solely a solution for Ethiopia; rather, it aligns with a global sustainability agenda. Framing the case of Addis Ababa within this wider context demonstrates both the universal applicability of waste‐to‐protein conversion and Ethiopia's significant capacity to participate in international trends in the circular bio‐economy. This comparative perspective reinforces the argument that fruit waste‐based SCP production is timely, practical, and globally significant.

### 4.2 Nutritional Composition of Fruit Waste

Fruit processing residues, particularly the peels, pomace, and pulp of bananas, mangoes, oranges, and other common tropical fruits, are rich in simple and complex nutrients, making them ideal substrates for the production of microbial biomass (single‐cell protein). In addition to residual starches and oligosaccharides that can be hydrolyzed into fermentable sugars, these wastes contain high concentrations of readily fermentable sugars (glucose, fructose, and sucrose) and soluble carbohydrates that serve as quick sources of carbon and energy for bacteria, filamentous fungi, and yeasts (Kim et al. 2024; Thiviya et al. 2022). Furthermore, the B‐group vitamins, minerals (K, Mg, Ca), and trace elements found in fruit peels and pomaces are essential micronutrients that lessen the need for costly supplements when growing SCP microorganisms (Li et al. 2024; Yasin et al. 2025). Dietary fiber, which includes insoluble cellulose/hemicellulose, soluble pectins, inulin, and resistant starch, is a second essential component. It serves as a large carbon reserve that can be converted to sugars by enzymes and promotes slower, sustained microbial growth in sequential or mixed‐stage fermentations (Aït‐Kaddour et al. 2024; Kim et al. 2024). Lastly, fruit wastes contain carotenoids and antioxidant polyphenols that can affect microbial physiology (metabolic fluxes, stress tolerance), and when properly managed, they can contribute functional value to downstream SCP biomass meant for feed applications (Thiviya et al. 2022; Yasin et al. 2025).

In contrast to many other organic substrates used for the production of single‐cell protein (SCP), fruit wastes (peels, pulps, and processing residues) are characterized by two important differences: they are usually rich in sugars that are easily fermented, simple soluble nutrients, and micronutrients that promote rapid microbial growth with little upfront hydrolysis, whereas common agricultural residues (e.g., straw, husks, and bagasse) are primarily composed of lignocellulose and need to be pretreated with energy‐intensive processes or saccharified by enzymes to release fermentable carbon (Thiviya et al. 2022; Kim et al. 2024). Accordingly, fruit wastes are appealing for decentralized valorization in urban environments such as Addis Ababa since they frequently allow for increased volumetric productivity and reduced processing complexity at small to medium scales (Sekoai et al. 2024; Li et al. 2024). In contrast, lignocellulosic residues, despite being much more plentiful and less expensive in bulk, have a lower immediate bioavailability and usually require more money, chemicals, or heat for delignification, which increases expenses and has an adverse effect on the life cycle unless they are incorporated into larger bio‐refinery schemes (Blasi et al. 2023; Ravindran et al. 2018). Lastly, from the perspective of application and product quality, SCPs made from fruit wastes can exhibit advantageous techno‐functional qualities (e.g., A. nutrient balances, water/oil holding, flavor profiles) and animal feed or ingredient use, but their higher moisture/acid content and seasonal and spatial variability necessitate careful feedstock handling and blending. In contrast, agricultural residues offer more stable year‐round supply chains, but process innovation is required to match the low processing footprint of sugary fruit streams (Dunuweera et al. 2021; Šelo et al. 2021).

### 4.3 Suitability of Fruit Waste for SCP Production

An abundant, underutilized feedstock, fruit wastes such as peels, pulp, seeds, and other processing residues are particularly pertinent to Addis Ababa's urban food system and market hubs, where organic material accounts for the majority of municipal solid waste and significant amounts of fruit residues are produced daily. Fruit wastes are readily available for little or no purchase price, require little pre‐processing for many microbial strains, and can substitute costly carbon sources (e.g., refined sugars) with single‐cell protein (SCP), making this conversion a low‐cost substrate pathway, reducing production costs significantly by these wastes (Li et al. 2024). Beyond economics, there are obvious environmental co‐benefits to using fruit waste to make SCP. It diverts high‐moisture organic wastes from informal disposal and landfills, which lowers the risks of methane and leachate. It also closes nutrient loops in urban food systems and, when evaluated using life‐cycle methods, can result in lower resource and greenhouse gas footprints than traditional protein sources. As part of integrated waste valorization and circular economy strategies, fruit waste is a compelling and feasible feedstock for developing SCP production in Addis Ababa due to its local abundance, strong substrate suitability, and observable environmental benefits (Fernández‐López et al. 2024).

The practical application of fruit waste as a feedstock for the production of single‐cell protein (SCP) in Addis Ababa is restricted by a number of issues. Firstly, the extremely high moisture content of fresh fruit by‐products (typically > 70%–90%) speeds up microbial spoilage and enzymatic degradation, necessitating quick processing or energy‐intensive drying/ensiling steps to prevent quality loss (Hasan et al. 2024). Second, untreated volumes usually deteriorate before they reach fermentation facilities because seasonal supply surges during harvest peaks create large, short‐term volumes that exceed local collection, transport, and processing capacity (Hasan et al. 2024). Third, when the final product is microbial biomass for feed or food, Ethiopia's unreliable cold chain and inadequate postharvest infrastructure make it challenging to maintain safe, uniform feedstock batches and raise the risk of contamination with opportunistic pathogens and spoilage organisms, which is a serious food safety concern (Etefa et al. 2022). Fourth, for yeast/bacterial SCP strains, the heterogeneity of fruit wastes (varying sugar, fiber, organic acid, and anti‐nutrient content between fruit types and seasons) makes process standardization difficult and may necessitate expensive pretreatment or nutrient supplementation to accomplish consistent yields. Lastly, the cost of turning fruit waste into SCP at a scale appropriate for Addis Ababa is increased by regulatory, logistical, and economic constraints (limited access to reasonably priced drying, storage, and decentralized fermentation capacity), which lowers competitiveness unless combined with focused investment and circular economy policies (Feleke et al. 2021).

This review sets itself apart from previous global analyses by zeroing in on the specifics of SCP production from fruit waste in Addis Ababa. Instead of broad generalizations, it digs into real, locally grounded data, considering actual waste volumes and the unique quirks of the city's infrastructure. Unlike studies that gloss over the details, this one places SCP feasibility squarely within the context of Addis Ababa's own market rhythms, seasonal fruit cycles, and the informal networks (like those around *Atikilt Tera* and *Shola*) that drive waste collection. These hubs consistently generate significant, concentrated fruit residues, making them ideal targets for microbial valorization. What's more, the review doesn't shy away from the city's particular challenges. There are clear constraints limited cold storage, unpredictable surges during harvest, and constantly shifting waste composition all of which affect everything from strain selection to process design. By mapping these Addis‐specific realities onto broader techno‐economic, environmental, and circular economy frameworks, the review delivers practical, city‐tailored insights for SCP implementation. In short, it bridges the gap between global theory and local practice, showing how SCP strategies can actually work in the dynamic, resource‐constrained context of urban Africa (Li et al. 2024; Sekoai et al. 2024; Fernández‐López et al. 2024).

## 5 Microorganisms and Fermentation Technologies for SCP Production

### 5.1 Common Microorganisms Used for SCP Production

**FIGURE 4:**
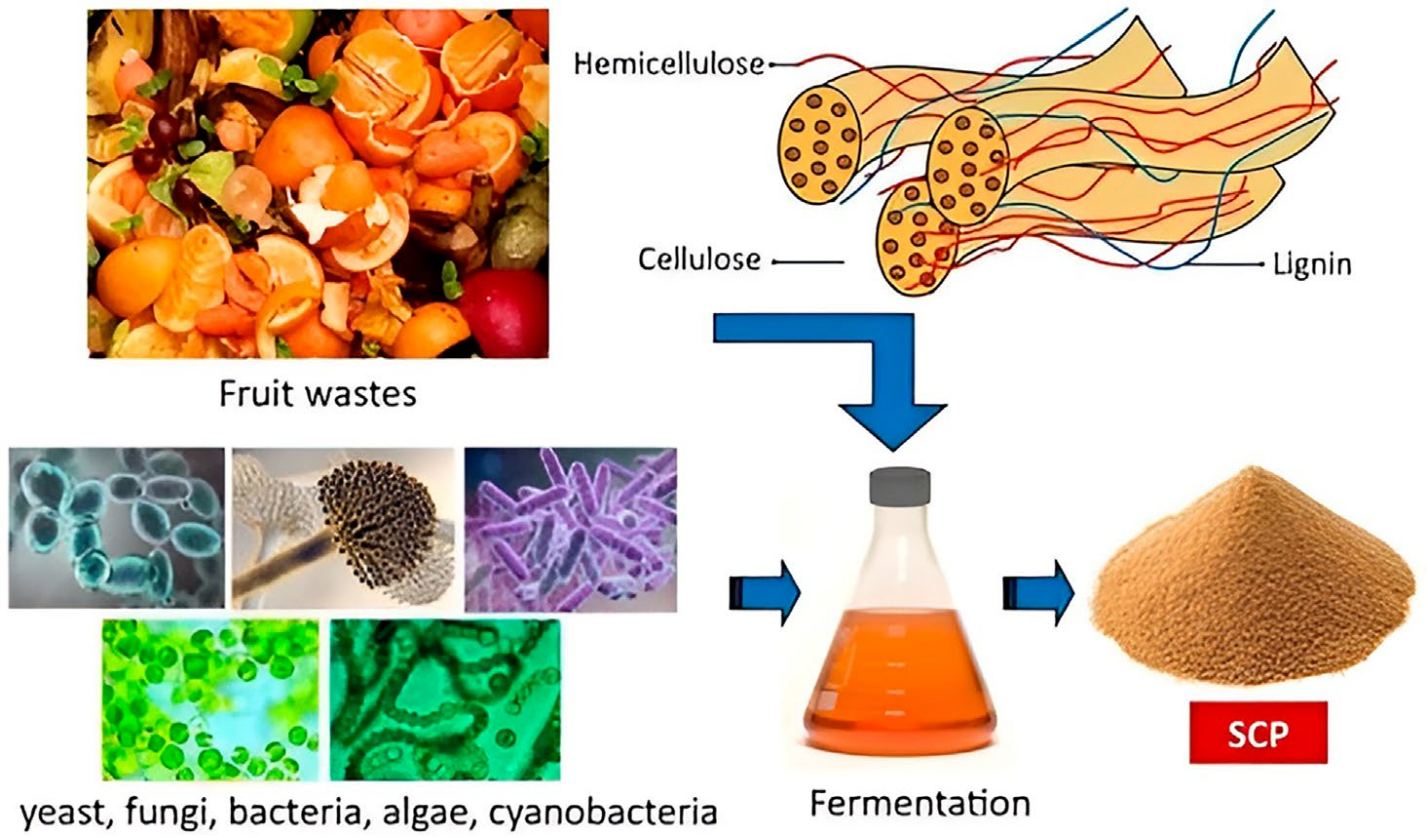
The common microorganisms used for SCP production.

In order to produce single‐cell protein (SCP) from fruit wastes, microorganisms should be chosen based on a variety of factors, including safety (history of food and feed approval), substrate range (capacity to metabolize sugars, pectins, and lipids found in peels and pomace), productivity (high growth rate and protein yield), cell composition (protein/RNA/ash), and downstream processing ease (harvesting and digestibility). In particular, *Saccharomyces cerevisiae, Pichia/Candida utilis (syn. Pichia jadinii), Yarrowia lipolytica, Kluyveromyces marxianus*, and *Pichia jadinii* continue to be the most viable options for fruit waste valorization, with certain filamentous fungi (e.g., such as *Rhizopus* species and *Aspergillus niger*) acting in specialized roles where cell wall‐degrading enzymes or pectinases are useful (Figure 4). The GRAS/QPS safety record, acid tolerance (good for low‐pH fruit residues), inhibitor tolerance, and ease of solid‐ liquid separation in comparison to bacteria and microalgae are the main reasons why yeasts are consistently ranked as the best in recent reviews (Koukoumaki et al. 2024; Sekoai et al. 2024; Li et al. 2024).

*Saccharomyces cerevisiae* is commonly used for SCP because it can withstand low pH and osmotic stress, grows quickly on mixed sugars that are present after mild hydrolysis of fruit peels or bagasse, and produces 40%–60% protein (dw) with balanced essential amino acids (lysine‐rich but frequently methionine‐limited). Most importantly, it makes use of established food/feed safety evaluations (QPS in the EU), which streamline regulatory processes for feed and food where RNA reduction techniques are used. The anchor organism for Addis Ababa's fruit‐waste context is *S. cerevisiae* due to its safety, robustness of the process, and worldwide strain availability (EFSA 2024; EESA 2025).

*Candida utilis* (*Pichia jadinii*) is distinguished by its high protein content, palatable flavor profiles, and effective biomass formation on pentose and residual fruit‐waste hydrolysates. Strong performance is reported by comparative studies at low pH and 30°C–35°C, which is consistent with the properties of many fruit side streams and lowers the risk of contamination. Its significance for waste‐to‐protein projects is maintained by its lengthy history in feed applications and regular inclusion in contemporary SCP overviews (Sekoai et al. 2024).

*Kluyveromyces marxianus* is food‐grade yeast that grows quickly and is thermotolerant. Because it can co‐utilize a variety of sugars, shorten fermentation cycles, and thrive at 40°C–45°C, *marxianus* is a desirable option for fruit wastes that have undergone minimal pretreatment. Recent research supports its safety positioning and shows promise for high‐rate SCP processes (QPS species, strain‐level assessment still required). Its thermotolerance is especially useful for enhancing process economics and lowering cooling loads in warm climates (Dong et al. 2025; Guo et al. 2025).

*Yarrowia lipolytica* is an oleaginous yeast that thrives on lipid‐rich residues (such as avocado and mango wastes) and mixed food waste slurries. It produces biomass that is high in protein and helps reduce COD, which is important for integrated wastewater/solid management. According to Yang et al. (2022), *Y. lipolytica* has established itself as a specialist in cases where fruit wastes contain oils or need strong inhibitor management due to recent high removals of organics with strong biomass productivity achieved through process intensification (e.g., two‐stage methods).

Filamentous fungi (*Aspergillus, Rhizopus*, and *Mucor*) are ideal for solid‐state fermentation (SSF) on peels and pomace because they can convert substrates that are rich in pectin and fiber through the use of secreted enzyme systems. Because of its industrial safety record, *Aspergillus niger* is commonly reported for SCP from citrus, pineapple, and orange wastes. However, strain selection is crucial and must remove aflatoxin makers ( *A. flavus* ), Studies employing a non‐aflatoxinogenic strain of *A. flavus* highlight the necessity of strict toxin monitoring when non‐GRAS species are taken into account, whereas recent work has demonstrated that *A. niger* can achieve high protein titers on fruit wastes in SSF/liquid systems. Overall, filamentous fungi can help downstream feed digestibility by producing SCP and pre‐digesting complicated fruit matrices when chosen appropriately (Ahmed et al. 2025; Shahzad et al. 2024; Khan et al. 2024).

In addition to the well‐established function of filamentous fungi and yeasts, current studies have brought attention to a number of additional microbial groups that have a great deal of promise for producing single‐cell proteins (SCPs), especially when it comes to the valorizing of fruit waste streams in Addis Ababa. First, because of their high protein content and quick growth rates, bacterial SCP producers are becoming more and more popular. For instance, purple phototrophic bacteria (PPB) are capable of producing biomass that contains up to 60% protein (dry cell weight, dw), enhanced with important nutrients such as polyhydroxyalkanoates (PHA), vitamins, carotenoids, and vital amino acids. These bacteria have the ability to break down organic waste and aid in the treatment of wastewater, providing both protein production and sanitation advantages (Zhuang et al. 2024). Methanotrophic bacteria, such as *Methylococcus capsulatus* , use methane as a carbon source and produce a remarkable 70%–80% dw protein content. Products such as FeedKind and UniProtein are currently on the market and are excellent alternatives to aquaculture feed (Zhuang et al. 2024). Another interesting option is *Clostridium* auto‐ethanogenum, an anaerobic chemo‐litho‐autotroph that produces biomass with a protein content of more than 80% dw by consuming hydrogen (H_2_) and carbon monoxide or dioxide (CO/CO_2_). According to Zhuang et al. (2024), this strategy also valorizes industrial gas emissions, as demonstrated by extensive operations in China.

The capacity of methylobacteria (such as *Methylobacterium* spp.) to use one‐carbon (C1) substrates like methanol and methane makes them emerging SCP workhorses. A recent review underscores their rapid growth, high protein yields, and versatility in SCP production, particularly for livestock and aquaculture feeds (Gundupalli et al. 2024). Microbial electro‐ synthesis (MES) is another innovative field that combines aerobic bacteria that consume acetate with anaerobic acetate production (by homoacetogens) to produce SCP directly from CO_2_ and electricity. In addition to achieving exceptionally high protein contents (~74% of dry weight) and biomass outputs of 17.4 g L^−1^ with an average production rate of 1.5 g L^−1^ day^−1^, a revolutionary bioreactor design also reduced effluent and increased sustainability (Pan et al. 2025).

Algal SCP producers in particular, cyanobacteria and microalgae remain important for combining protein synthesis and waste valorization. It has been demonstrated that species like *Chlorella* and *Scenedesmus obliquus* can produce up to 52% SCP content from a variety of wastewater streams, including industrial effluents, tofu, and tempeh (Bratosin et al. 2021). *Spirulina* (*Arthrospira* spp.) is a promising candidate for resource‐integrated protein production systems because it produces 48%–56% SCP (dw) when cultivated in salty or desalinated wastewater (Bratosin et al. 2021). In addition to protein, microalgae provide bioremediation benefits by eliminating organic loads, nitrogen, phosphorus, and even pathogens from wastewater. This is especially important in situations involving decentralized fruit processing (Le and Nguyen 2024). Finally, SCP is produced by gas‐fermenting bacterial systems, including *Cupriavidus necator* , which directly utilize hydrogen (H_2_) and CO_2_ through gas fermentation. Continuous fermentation techniques have been used recently to increase yields, and customized bioreactor designs that increase daily biomass output hold promise for turning CO_2_ emissions into microbial proteins with added value (Fu et al. 2025).

### 5.2 Fermentation Process Overview

Successful SCP production begins upstream with clean substrates and a robust inoculum. When it comes to nutrient‐rich fruit hydrolysates, aseptic handling and vessel sterilization are crucial in preventing wild microorganisms from outcompeting produced strains. Reviews always emphasize axenic conditions and clearly described upstream procedures (media preparation, broth and gas sterilization) before bioreactor transfer (Ye et al. 2024; Kumar, Raj, et al. 2024). In order to attain high cell viability and physiological preparedness (late‐ exponential phase) prior to pitching, inoculum development usually takes place in seed flasks. Although the organism and substrate determine the optimum inoculum size, larger inoculation rates can boost peak cell dry weight and decrease lag phases. This effect has been seen in a variety of SCP systems, including current gas‐fermentation experiments. Several investigations use a v/v inoculum of several percent to quickly dominate the mixed sugars generated from fruit wastes, such as peels and pomace (Ye et al. 2024; Fu et al. 2025).

Fermentation time must balance biomass yield, protein content, and operational economy. *Saccharomyces cerevisiae* and other yeasts typically reach their maximum biomass/protein on fruit‐waste hydrolysates in 48–96 h, whereas autolysis and nutrient constraint might subsequently degrade quality. Protein peaks, for instance, around days three and four for banana‐peel media, highlighting the significance of monitoring the development phase as opposed to operating arbitrary extended batches. *S. cerevisiae* on pineapple waste media has also generated gram‐per‐liter SCP in brief batch windows. According to Azwar et al. (2021) and Koukoumaki et al. (2024), this kinetics supports time‐resolved monitoring (optical density, dry weight, residual sugars) in order to determine the actual harvest point for any local waste stream. From a sustainability perspective, LCA evaluations of yeast‐based SCP on agri‐side streams have shown that shorter, strategically timed batches reduce energy use and downstream loads (Fernández‐López et al. 2024).

Although temperature is one of the most powerful factors influencing growth rate and protein yield, species‐specific factors determine the ideal set point. Many investigations on *S*. *cerevisiae* operating with mixed fruit wastes operate at temperatures close to 30°C, with pH values of about 4.5 and active mixing/aeration to maintain the availability of sugars and oxygen. Conditions such as 30°C, ~pH 4.5, 300 rpm agitation, and moderate airflow have facilitated rapid yeast growth and conversion in the simultaneous saccharification–fermentation of mixed food wastes (Tropea et al. 2022). Different control windows are required by other production hosts: While *Pichia pastoris* exhibits growth and expression optima around 30°C with performance dropping above ~32°C, *Kluyveromyces marxianus* can tolerate warmer temperatures and can speed up processes where cooling is expensive, highlighting the need for strain selection and local utility costs to co‐inform temperature strategy (Koukoumaki et al. 2024; Cos et al. 2006).

In order to convert heterogeneous streams into consistent protein, it is important to get the start (inoculation), schedule (time), and set point (temperature) right. Fruit wastes (banana, citrus, mango, pineapple peels/pulp) offer fermentable sugars and micronutrients, but they differ in sugar release and inhibitors. In resource‐constrained settings, careful control of these fundamentals, along with pH, aeration, and agitation, drives both yield and quality while minimizing contamination and energy overheads, according to recent syntheses of SCP progress and fruit‐waste utilization (Thiviya et al. 2022; Bajić et al. 2022; Li et al. 2024). For microbial cultivation targeted at producing single‐cell protein, fruit wastes high in fermentable sugars, pectin, cellulose, and hemicellulose are attractive substrates (Thiviya et al. 2022). Pre‐treatment is necessary to release fermentable sugars because the structural complexity of lignocellulosic components (cellulose, hemicellulose, lignin, and pectin) limits microbial access and hydrolysis (Table 1). Furthermore, to avoid antimicrobial effects on fermenting bacteria, some inhibitory chemicals, such as limonene in citrus peels, must be eliminated during pre‐treatment (Thiviya et al. 2022).

**TABLE 1:** Summarizing the pretreatment and hydrolysis approaches for fruit waste fermentation efficiency enhancement.

Pre‐treatment/Hydrolysis Method	Key findings	Advantages	Limitations/considerations	Recent sources
Hydrothermal (135°C, pH 5, 40 min)	> 40 g reducing sugars/100 g waste (~97% carbohydrate recovery), > 95% fermentation efficiency	High sugar yield, minimal inhibitors	Requires heat and pH control equipment	Sánchez et al. (2023)
Thermal pretreatment	SCOD increases 57%, higher VFAs, faster digestion, and gas yield increases 6.5% (~767 mL/g)	Enhance solubilization and digestibility	Focused more on biogas than SCP	Sun et al. (2024)
Two‐stage digestion (pre‐hydrolysis + mesophilic)	68.5% volatile solid removal, high biodegradability of banana peel waste	Improve microbial access and conversion	Longer process and scaling challenges	Odedina et al. (2017)
Dilute sulfuric acid (DSA)	Boosted Euglena graccilis cell density (1.35 times), β‐1,3‐glucan yield increase (12.9%–24%)	Strong sugar release, improved fermentation	Neutralization step needed, equipment corrosion	Zhu et al. (2024)
Biological (Microbial Delignification)	Pleurotus, Trichoderma, Bacillus degraded lignin, improving enzymatic hydrolysis	Eco‐friendly, targets lignin barrier	Slower processing, culture maintenance needed	Zhang et al. (2021)
Enzymatic hydrolysis (General)	High monosaccharide yield, especially glucose, from fruit residues	Selective, high digestibility	Costly enzymes, pretreatment still required	Khantibongse and Ratanatamskul (2023)

### 5.3 Optimization of SCP Production

Optimizing SCP output from fruit‐waste substrates rests on precisely regulating conditions for cultivation and substrate quality (Table 2). The biomass productivity of yeasts commonly used on peel/pulp hydrolysates (like *S. cerevisiae* ) usually peaks at mildly acidic pH ~4.5–5.5 and 28°C–33°C, which also suppresses contaminants. However, thermo‐tolerant strains and stressed systems can shift optima (growth maxima reported near pH 7.0, 40°C in saline, non‐detoxified hydrolysate, for example), highlighting the necessity of strain–substrate matching. High cell yields and respiratory growth depend on adequate aeration/agitation (oxygen transfer, kLa), whereas inoculum size (typically 5%–10% v/v) reduces lag and enhances uniformity. Supplementing with micronutrients (Mg, Zn, Fe), vitamins, and easily assimilated nitrogen (ammonium, urea) results in a balanced C/N ratio, prevents restrictions, and increases protein content (Sekoai et al. 2024). Sugar release is improved and inhibitors are lessened on the substrate side by pretreatment and (if necessary) detoxification (enzymatic/acid hydrolysis, limonene or phenolics removal in citrus/banana peels); fed‐batch or pH‐stat control helps prevent acid crash as organic acids build up. Choice of strain is important: Mixed cultures can co‐utilize sugars, and *K. marxianus* and *Y. lipolytica* can outperform *S. cerevisiae* on specific wastes and endure heat or lipids (Lucaroni et al. 2022). Last but not least, process mode affects yield. SSF on moist peels uses less water and energy and can increase volumetric productivity, while SmF provides easier scale‐up and tighter control. The most dependable method for achieving the highest SCP production is a design‐of‐experiments strategy that maps the pH, temperature, C/N, and aeration interactions on the particular Addis Ababa fruit‐waste mix (Thiviya et al. 2022).

**TABLE 2:** Recent studies on the optimization of SCP production (substrate, microbes and optimization).

Substrate	Microorganism(s)	Optimization approach	protein yield/biomass	References
Mixed fruit and vegetable waste (orange peel, onion peel and pea pods)	*Lactobacillus sp*	Submerged fermentation (SmF), tested pretreatment and nutrient supplementation	Biomass yield 10.4 g/L; crude protein ~41% (w/w)	Kaur and Chavan (2022)
Banana peel (and other food waste)	*Saccharomyces cerevisiae* (and mixed culture)	SmF, aeration, pH, C/N and pretreatment and optimization (DoE/RSM in follow)	12.8 g/L dry biomass; protein content 45%–48%	Khan et al. (2022)
Various fruit wastes (papaya, oranges etc.)	Mixed culture/natural *toddy consortia*	Comparative substrate trials; nutrient supplementation and incubation mode	Biomass productivity 9–11 g/L; protein ~42%	Thiviya et al. (2022)
Dairy/fermentable sugar waste	*Kluyveromyces marxianus* (new isolates)	Fed‐batch and batch comparisons; pH, temp, aeration, strain selection	Fed‐batch: 15.6 g/L biomass; protein ~50%; batch: 11.2 g/L	Koukoumaki et al. (2024)
Vegetable wastes (tomato, capsicum, eggplant)	Filamentous fungi	Solid‐state fermentation optimization; moisture, pH, incubation time (RSM)	SCP yields 180 mg/g dry substrate; crude protein 38%–40%	Shahzad et al. (2024)
Organic acid rich waste and hydrolysates	*Saccharomyces cerevisiae*	Studied the impacts of organic acids, nitrogen forms and ionic species	Biomass 10.1 g/L; protein 44%	Zeng et al. (2022)
Survey across substrates	Survey across microbes	Synthesis strain choice, pretreatment, incubation mode, feed‐batch vs. SSF, metabolic engineering and process integration	Ranges reported: 8–16 g/L biomass; protein 40%–55% (meta‐analysis)	Li et al. (2024); Sekoai et al. (2024) (recent review and meta‐analysis)

Fruit processing residues, such as orange, banana, papaya, and mixed fruit peels and pomaces, have been shown in a number of recent laboratory and pilot‐scale studies to be efficient substrates for the production of microbial biomass (single‐cell protein, or SCP) when combined with yeasts, filamentous fungi, or mixed microbial cultures. Orange residues can be used as the only carbon source for SCP, as demonstrated by the significant biomass and protein produced by *Candida utilis* grown on orange‐peel hydrolysates following process optimization (Carranza‐Méndez et al. 2022). Banana peel one of the most prevalent tropical fruit wastes has repeatedly been converted to SCP utilizing Saccharomyces and filamentous fungi with documented improvements in crude‐protein production and acceptable proximate composition after simple pre‐treatments (acid/heat or enzymatic hydrolysis). Banana‐peel derived SCP shows promise as a poultry/animal feed supplement, according to pilot research and current experimental work (Khan et al. 2024; Azwar et al. 2021). Regionally available peels and mixed fruit wastes have also been successfully valorized: in multiple studies, multi‐waste submerged fermentation trials (banana, citrus, carrot pomace, and potato peel) and papaya peel fermented with native toddy/mixed cultures produced protein‐rich biomass suitable for feed trials, highlighting the adaptability of SCP processes to local feedstock mixtures (Thiviya et al. 2022; Khan et al. 2022).

The findings of these case studies all point to three useful conclusions: (1) the highest biomass/protein is produced by simple pre‐treatment (to release fermentable sugars) combined with the selection of robust microbial strains; (2) SCP from fruit waste consistently improves waste valorization and provides promising nutritional profiles for animal feed; and.

(3) The main obstacles to widespread adoption are still techno‐economic and safety issues (scaling, nucleic acid reduction, mycotoxin/contaminant control, and uniform feedstock supply). The best ways to get over these restrictions, according to recent assessments, are through strain/bioprocess engineering and process integration (hydrolysis, fermentation, downstream drying) (Sekoai et al. 2024; Li et al. 2024). Implications for Addis Ababa: these global case studies demonstrate the feasibility of turning common local residues (banana and citrus peels, carrot bagasse, and mango residues) into SCP using simple technology (submerged fermentation + hydrolysis) and producing valuable protein for animal feed. However, to de‐risk commercialization lessons directly derived from the listed case studies and reviews should specifically address local scaling, supply‐chain seasonality, low‐ cost pre‐treatment options, regulatory/safety testing, and pilot demonstrations (Khan et al. 2022; Sekoai et al. 2024).

Contrary to the prevailing emphasis on *S. cerevisiae* in fruit‐waste hydrolysate research, its performance isn't uniformly outstanding. For example, Khan et al. (2022) reported that *S. cerevisiae* yielded approximately 12.8 g/L biomass with 45%–48% crude protein from banana peel hydrolysates. In contrast, Koukoumaki et al. (2024) achieved notably higher yields of about 15.6 g/L biomass and 50% protein using *K. marxianus* under fed‐batch conditions. This suggests that thermo‐tolerant, stress‐resistant yeast strains may surpass conventional strains, particularly when processing inhibitor‐rich, non‐detoxified substrates. Meanwhile, Thiviya et al. (2022) observed that mixed microbial consortia produced around 11 g/L biomass, outperforming single strains in productivity. However, such consortia introduce issues with reproducibility and contamination, complicating large‐scale applications. These varying results underscore a persistent challenge in optimizing strain substrate combinations, and indicate that no universally optimal microbial platform exists for all fruit‐waste streams. A further limitation involves the scalability and practical feasibility of these optimization strategies. Although fed‐batch and nutrient‐enriched systems reliably maximize protein yields in controlled laboratory settings (Li et al. 2024; Sekoai et al. 2024), their operational costs and technical demands restrict their applicability in settings with limited infrastructure, such as Addis Ababa. In such contexts, solid‐state fermentation on mixed fruit peels may be more viable, given its lower water and energy requirements even if it sacrifices some volumetric control. Recent urban waste audits indicate that approximately 70%–74% of Addis Ababa's municipal waste is biodegradable, with fruit and vegetable residues concentrated in areas like Atikilt Tera (CSA 2023; Gelan 2021). Therefore, optimizing processes for this environment necessitates prioritizing robust, flexible microbial strains, cost‐effective pretreatment methods, and simplified operational models over high‐yield, capital‐intensive systems.

## 6 Potential of Fruit Waste for SCP Production in Addis Ababa

### 6.1 Current Fruit Waste Management Practices

Fruit and other organic waste from homes and businesses in Addis Ababa is still primarily disposed of in municipal landfills rather than being systematically valued. With only a small portion going into formal recycling or treatment streams, the majority of municipal solid waste (MSW), including fruit and vegetable residues from homes and markets, is collected and sent to landfills or open dump sites (formerly the Koshe site, which is currently the focus of waste‐to‐energy and remediation projects). Due to this, a lot of biodegradable garbage is landfilled or thrown in the open, which has negative effects on the environment and public health (Teshome 2021). Small‐scale composting and new private initiatives are beginning to collect some organic fractions for soil amendment and urban agriculture in addition to landfilling; nevertheless, organized organic diversion and citywide source separation are still few. Academic and pilot programs have demonstrated the obvious composting potential of source‐separated organics; however, institutional, logistical, and technological obstacles (cost, contamination, collection, and restricted market connections) stand in the way of mainstreaming composting (Obsa et al. 2022).

A significant amount of fruit waste is also unofficially redirected to animal feed or consumed by scavenger populations. Fruit peels and pulps are frequently used by market vendors, peri‐ urban livestock keepers, and unofficial feed routes (typically after minor processing such as drying or chopping). These unofficial uses lessen the amount of waste that ends up in landfills, but they also bring up concerns about food safety, seasonality, and supply fluctuations, all of which restrict trustworthy large‐scale valuation (Duguma et al. 2024). There is a clear opportunity to reroute high‐carbohydrate fruit residues toward controlled bioprocesses like single‐cell protein (SCP) production if collection and pre‐treatment (segregation, drying/chopping, contamination control) can be improved. This is because the current system concentrates fruit waste in mixed MSW streams and only partially in informal feed/composting channels. However, scalable SCP valorization will require investment in source separation, cold‐chain or rapid processing for perishable wastes, and quality controls to meet bioprocess feedstock specifications (Ali et al. 2024).

Due to inadequate funding and ill‐coordinated collection, segregation, and recovery methods, Addis Ababa's urban waste management is still primarily unsustainable. Massive amounts of mixed municipal solid waste are frequently dumped in open dumps or unmanaged landfills instead of being recovered or valorized due to rapid urbanization and growing organic waste generation that exceeds municipal capacity. This results in ongoing logistical, environmental, and public health risks (Gebrekidan et al. 2024; Adefris et al. 2023). Limited source separation, inadequate enforcement of policies, minimal community involvement, and insufficient investment in processing infrastructure (composting, anaerobic digestion, or bioconversion) are the main causes of inefficiencies. These disparities result in losses due to spoiling and contamination (plastic, soil, medical waste), which makes biological valorization challenging or dangerous. They also raise transportation and disposal costs and lower the caloric/nutrient quality of organic streams available for value‐added uses (Debele and Fereja 2024; Taye et al. 2024).

The current state of affairs misses a number of opportunities from a sustainability perspective. For example, recovering high‐value fruit and vegetable by‐products for controlled bioprocessing (e.g., single‐cell protein, compost, or bioenergy) would reduce methane and leachate from dumpsites, conserve resources, and create local feed/food inputs. However, this requires investment in segregation at the source, cold‐chain or rapid stabilization for high‐moisture wastes, clear regulatory pathways for novel products, and capacity building for informal actors in the waste stream. The conversion of fruit waste from Addis Ababa into SCP is theoretically promising, but it depends on addressing systemic inefficiencies in terms of contamination control, policy/institutional support, and collection/segregation (Shimelis et al. 2024; Sekoai et al. 2024).

### 6.2 Prospects for Integrating SCP Production

An abundant disposal issue is transformed into a feedstock advantage by using fruit waste in the manufacture of single‐cell protein (SCP). Because fruit peels and processing residues are high in fiber, micronutrients, and fermentable sugars, they are ideal for promoting rapid microbial growth. Using these substrates keeps organic matter out of landfills and lowers the risks of leachate and greenhouse gas emissions that come with traditional disposal. In addition to lowering municipal and industrial waste management expenses, this waste‐to‐feedstock loop also aligns SCP routes with the objectives of the circular economy (Sekoai et al. 2024; Pal et al. 2024). Fruit‐waste‐based SCP provides an inexpensive, regionally scalable source of high‐quality protein from an economic and food security standpoint. In urban and peri‐urban areas like Addis Ababa, where fruit processing produces consistent amounts of peel and pulp waste, microbial biomass cultivated on fruit residues can achieve protein contents and amino‐acid profiles comparable to conventional feeds while requiring far less land, water, and time than animal or plant protein systems. Producers can lessen their reliance on imported feed proteins and establish new value chains for smallholders and processors by converting locally accessible residues (such as banana, citrus, and carrot) into protein (Li et al. 2024; Thiviya et al. 2022). Lastly, integrating fruit waste into SCP production promotes technological and social co‐benefits: it unlocks co‐products (fibers, antioxidants) that increase overall profitability, supports innovations in preprocessing and strain selection to maximize yield and safety, and promotes modular, small‐scale fermentation units that can operate close to waste sources (reducing transport emissions). Early lifecycle and techno‐economic studies demonstrate encouraging decreases in production costs and environmental impact when SCP employs food industry residues, which makes fruit waste valorization an appealing tactic for Ethiopia's sustainable protein expansion (Fernández‐López et al. 2024; Khan et al. 2024).

True economic feasibility in Addis Ababa depends on the entire value chain, including collection/segregation, pre‐treatment, fermentation, downstream processing, and market placement, even if fruit waste provides an inherently inexpensive substrate for the creation of single‐cell proteins (SCPs). Although there is a plentiful source of feedstock due to the city's high production of organic‐rich MSW (household and market fruit/vegetable residues), the low segregation rates and existing mixed‐waste collection systems increase the costs of contamination and transportation, which must be factored into any business plan. Feedstock cost and unpredictability can be significantly decreased by strengthening source separation and collaborating with markets/processing hubs (Hirpe and Yeom 2021; Kassahun et al. 2025). Scale and technology have a significant impact on SCP plant capital and operating costs. For feed‐grade SCP, small decentralized fermenters reduce transportation costs and enable SMEs to make incremental investments, while larger centralized facilities benefit from economies of scale but demand higher upfront capital and consistent, steady feedstock streams. By boosting productivity per reactor volume and lowering energy consumption, process intensification and two‐stage or integrated fermentation techniques which are said to increase yields and decrease residence time improve unit economics. Biogas, compost, or soluble nutrient streams are examples of co‐product valorization that increases returns and lower waste management expenses. Profitability depends on process improvement and integration with current organic‐waste valorization (such as biogas or compost schemes), according to recent SCP techno‐economic assessments and practical research (Li et al. 2024; Yang et al. 2022).

In terms of pricing and nutritional content, SCP for animal feed must contend with regionally accessible protein sources (fishmeal, soybean meal). Locally, Ethiopia's high organic‐waste fractions and relatively low labor costs are advantageous, but unclear regulations, a lack of funding for agro‐industrial SMEs, and weak product acceptance channels are major obstacles to scale. International studies indicate that emerging protein technologies, such as e‐protein and advanced SCP, have the potential to reach competitive cost levels if energy and process efficiencies improve. Private sector off‐takers (poultry/fish feed mills), public incentives (waste diversion objectives, subsidies for waste‐to‐value SMEs), and pilot demonstration projects in Addis Ababa might all help close the “first‐mile” gap and reduce investment risk. Small to medium‐sized SCP operations integrated into existing waste‐management/value‐chain hubs demonstrate reasonable payback periods and positive net benefits when conservatively modeled, taking into account collection and preprocessing, capital amortization, and modest yield improvements. This is particularly true when co‐products and avoided landfill costs are taken into account. However, before full commercial deployment, thorough, site‐specific techno‐economic assessments and pilot trials are advised (Fasihi et al. 2025; Buchner et al. 2024).

### 6.3 Local Infrastructure and Knowledge

The municipal infrastructure of Addis Ababa offers both a potential and a constraint for the production of single‐cell protein (SCP) from fruit and vegetable waste. The city produces a large amount of easily fermentable, organic waste (often more than 70% biodegradable), which makes it a good feedstock for SCP processes. Nevertheless, the current collection systems are still dispersed, with little source separation and significant informal‐sector handling, which lowers feedstock consistency and raises the risk of contamination (Gebrekidan et al. 2024; Sisay et al. 2024). There are currently not many pilot‐to‐demo‐scale fermentation facilities in the city specifically designed to turn food waste into microbial biomass. The majority of bioprocessing capacity is located in research facilities and industrial parks that were initially built for small‐scale biotechnology or agro‐processing; these resources could be expanded or repurposed for the production of SCP, but reliable scaling will require focused investment (bioreactors, downstream drying/packaging, sterilization equipment, etc.) and technical collaborations (Fanuel et al. 2022; Li et al. 2024).

Underdeveloped cold‐chain and logistics requirements for safe SCP handling and distribution include irregular collection schedules, a dearth of sanitary transfer stations, and little on‐site preprocessing (heat treatment, ensiling, or size reduction), which raises the risk of microbial contamination and spoiling before fermentation. The implementation of source segregation at markets and processing facilities, the strengthening of transfer‐station sanitation, and the deployment of small mobile preprocessing units would significantly increase usable feedstock yields and stabilize the supply for fermenters (Fanuel et al. 2022; Gebrekidan et al. 2024). SCP distribution at the market level is hampered by value‐chain, quality‐assurance, and regulatory issues. Ethiopia currently lacks clear national standards and commercial distribution channels for microbial biomass, so the adoption of SCP for animal feed (aquaculture/livestock) is contingent upon its proven safety, consistent nutritional profile, and affordable packaging/transport areas. To generate demand and a dependable off‐take conduit, public‐private partnerships, demonstration farms, and coordination with feed regulators will be crucial (Sekoai et al. 2024; Buchner et al. 2024).

The local industry exhibits glaring knowledge and capacity gaps that restrict the conversion of fruit processing leftovers into SCP, despite the fact that there are plenty of these residues in Addis Ababa. Due to a lack of local expertise in second‐generation feed stocks and SCP process design (substrate pre‐treatment, controlled fermentation, nucleic acid reduction, and downstream biomass processing), many appropriate technologies are either imported as turnkey solutions or remain at lab scale instead of being locally adapted systems. The technical difficulty of valuing diverse fruit wastes and the requirement for process development according to the situation are highlighted in recent reviews on food waste SCP (Sekoai et al. 2024; Li et al. 2024). Second, there are reports of a lack of human resources and skills in Ethiopia's agro‐processing sector. Current TVET and industry training programs underrepresent process engineering, microbial fermentation control, quality assurance (food safety/HACCP), and product formulation for human consumption and animal feed. The technical requirements of contemporary agro‐processors, particularly in Addis Ababa's MSME clusters, are not adequately met by the TVET curricula that are currently offered, according to national assessments and policy studies. Uptake would be accelerated by bolstering short‐course modules and vocational training centered on fermentation biotechnology, bioprocess monitoring, and lab–to–pilot scale translation (Stock 2025). Third, scale‐up is hampered by institutional and cross‐sectoral knowledge gaps: poor connections among academic institutions, research centers, and private processors delay the commercialization of pilot results (such as local strain selection and substrate optimization). In order to guarantee sustainability and uptake, recent feasibility studies on innovative SCP deployments in Ethiopia specifically advise combining trial installations with integrated capacity building (technical, regulatory, and commercial skills) (Buchner et al. 2024).

## 7 Challenges and Barriers to SCP Production From Fruit Waste in Addis Ababa

### 7.1 Technological Challenges

Fruit‐processing residues for single‐cell protein (SCP) have shown encouraging results, but a number of biological and technological limitations impede the product's transition from laboratory to market. In order to guarantee regular fermentations, the seasonality and substrate heterogeneity of fruit waste (varying amounts of sugar, moisture, and inhibitors) necessitate intensive pretreatment and feedstock conditioning, which raises complexity and costs (Dunuweera et al. 2021; Sekoai et al. 2024). Second, there are antimicrobial compounds (phenolics, organic acids) and particulate solids in many fruit wastes that hinder growth or necessitate hydrolysis or clarification; research into efficient, affordable pretreatments (mechanical, diluted acid, or enzymatic) is still ongoing (Kim et al. 2024).

On the microbial side, widely used yeasts and fungi provide good yields but have disadvantages, such as strain‐specific substrate ranges, high nucleic acid content (which necessitates expensive reduction steps for human food), and the possibility of yeasts or molds carrying allergens or mycotoxins if not properly chosen and monitored. Although yields and substrate flexibility can be increased through genetic and metabolic engineering, modified strains encounter difficulties with public acceptance, stability at scale, and regulatory barriers (Jach et al. 2022; Zhuang et al. 2024). Limitations in engineering and scaling up also remain: process control systems and downstream separation (biomass harvesting, drying, and decontamination) continue to be significant cost drivers that undermine SCP's economic advantage; submerged and solid‐state reactors behave differently with high‐solid, sticky fruit residues, making mass/heat transfer, oxygenation, and contamination control more difficult (Koukoumaki et al. 2024; Li et al. 2024). Given these limitations, it is clear that achieving SCP from fruit waste in Addis Ababa (or comparable urban settings) will necessitate a combination of robust, low‐cost pretreatments, safety‐focused strain selection and engineering, and customized bioreactor designs that minimize downstream costs, all of which are backed by techno‐economic and life‐cycle analyses (Sekoai et al. 2024; Fernández‐López et al. 2024). Addressing safety in single‐cell protein (SCP) production from fruit waste isn't just about nucleic acids; issues like allergenicity and mycotoxin contamination are equally significant. Certain yeasts and filamentous fungi thriving on improperly sanitized substrates or under poor storage can churn out allergenic proteins or dangerous secondary metabolites like aflatoxins and ochratoxins. That's a real risk, not some distant hypothetical. To counteract this, fundamental steps such as substrate sterilization, strict fermentation controls, and thorough post‐harvest decontamination are absolutely crucial. Integrating robust quality monitoring—think rapid mycotoxin tests and allergen screening—should be a non‐negotiable part of any SCP production line. Bringing these safety protocols together with smart strain selection and process optimization does more than tick boxes for regulatory compliance (Koukoumaki et al. 2024). It builds public trust, which is critical if SCP is going to take off as a sustainable protein source in urban environments like Addis Ababa. Without this kind of diligence, the whole system risks losing credibility before it even gets off the ground.

A significant barrier to commercialization for the large‐scale synthesis of SCP from fruit waste is the absence of standardized procedures. Without strong, standardized feedstock specification and preprocessing protocols, fruit waste's composition (sugars, acids, moisture, and inhibitors) varies greatly depending on the fruit type, season, and processing stream. This compromises reproducible fermentation performance (Sekoai et al. 2024; Li et al. 2024). There are no commonly recognized standard operating procedures (SOPs) for substrate conditioning (e.g., washing, size reduction, enzymatic hydrolysis, or sterilization), inoculum preparation, bioreactor operating windows (aeration, pH, and temperature), or downstream drying and detoxification specific to fruit‐derived substrates at the process level. As a result, pilots that report high lab yields frequently do not translate to large‐scale production. According to Zhuang et al. (2024) and Ritala et al. (2017), this fragmentation raises the danger of contamination, product variability (protein content, nucleic acid level, ash, and antinutrients), and regulatory uncertainty for feed and food purposes.

### 7.2 Economic and Market Barriers

According to recent techno‐economic analyses, the cost of single‐cell protein (SCP) varies greatly depending on the organism and feedstock, but it is generally declining. Using food or fruit waste as a substrate can reduce production costs by approximately 35%–75% when compared to refined sugars because the waste is inexpensive or negative and part of the total costs is driven by the substrate (Salazar‐López et al. 2022; Li et al. 2024). According to Jiang et al. (2024), Rovira‐Alsina et al. (2025), and Moscariello et al. (2025), the cost of hydrogen/bio electrochemical routes is still ~$19/kg for hydrogen‐oxidizing bacterial systems and $1–1.7/kg in favorable electro‐ or waste‐based scenarios. This indicates that there is a significant amount of headroom as scale and power prices improve. In contrast, plant proteins are still reasonably priced on the market: in late 2023–2024, soy protein isolate averaged around US $2.5–2.7 per kilogram worldwide, whereas in Addis Ababa, Ethiopia, retail soy flour typically sells for US $2.4–3.4 per kilogram (Wale 2024). Locally, traditional animal proteins are much more expensive per kilogram of product; in 2025, Addis Ababa retail beef will normally cost between US$6.1 and US$7.8/kg (Abebe et al. 2022). According to recent reviews that highlight agricultural‐waste substrates as a cost‐effective route for SCP, SCP made from fruit and vegetable wastes has a credible path to undercut meat on a price basis and approach soy on a per‐kg product (and possibly per‐kg protein) basis as scale, continuous fermentation, and low‐cost waste valorization mature (Li et al. 2024).

Single‐cell protein (SCP) produced from fruit waste is still relatively new in Addis Ababa, but it has great potential. Price, health, and environmental narratives have sparked consumers' cautious interest in alternative proteins, but there is a lack of general familiarity and trust. Therefore, uptake will require targeted education, clear labeling, and sensory testing (Malila et al. 2024; Gençer Bingöl and Ağagündüz 2025). Due to operational and supply‐chain obstacles (collection, pre‐treatment, cold‐chain, and quality assurance) that hinder early adoption, public‐private pilot projects and incentives to reduce investment risk are essential for industry actors such as food processors, animal feed manufacturers, and waste collectors (Debele and Fereja 2024; Nath et al. 2023). In terms of policy, Ethiopia has begun to specify priorities for the bioeconomy and waste valorization (draft national strategies and donor‐supported programs). This has opened up a window for regulatory pathways, safety/traceability standards, and financial support that, when combined with stakeholder engagement, would hasten the commercialization of SCP. In order to scale fruit waste‐to‐SCP pathways in Addis Ababa, it will be helpful to align consumer attitudes, industrial practices, and policymaker agendas by framing SCP within circular bioeconomy, climate, and food security goals and showcasing clear nutritional, safety, and economic advantages through locally visible pilots (Pereira et al. 2022).

### 7.3 Environmental and Social Considerations

Producing single‐cell protein (SCP) from fruit waste can benefit local communities like Addis Ababa in the short term by generating jobs along the waste‐to‐protein value chain, including collecting and sorting peels and pulp, preprocessing (drying, milling, hydrolysis), fermentation, quality control, and distribution. Additionally, it can integrate and upskill informal waste workers into safer, higher‐paying positions in the expanding circular bio‐ economy sector. Particularly in the teenage labor market of Sub‐Saharan Africa, recent evaluations of food‐waste‐to‐SCP emphasize not only environmental benefits but also local entrepreneurship potential for micro and small firms (Sekoai et al. 2024; Okuthe 2024). SCP can enhance public health beyond employment by lowering pathogen exposure linked to unmanaged organic waste and by providing high‐quality, reasonably priced protein and micronutrients in areas where undernutrition is persistent. Ethiopia's most recent national survey continues to report significant child stunting and undernutrition among women, highlighting the need for easily accessible protein sources (Woldeyohannes et al. 2023). Human research also shows that myco‐protein, a popular SCP, has health benefits. Randomized trials have shown that replacing meat with myco‐protein lowers LDL cholesterol and improves cardio‐metabolic markers. This suggests that if SCP foods are made widely available, there will be benefits for the entire community (Pavis et al. 2024; Farsi et al. 2023). An approach that is directly applicable to fruit‐waste‐to‐SCP hubs in Ethiopian cities is the conclusion drawn by circular‐economy analyses and development briefs that, when combined with decent‐work safeguards, waste valorization can be labor‐intensive and job‐creating across the value chain (Khanna et al. 2024; Holmberg and Ideland 2021).

Large‐scale fruit waste recycling into single‐cell protein (SCP) can provide significant environmental benefits by eliminating water‐ and land‐intensive animal or soy proteins and diverting high‐moisture organics from landfills and dumpsites, which are important producers of methane in rapidly expanding cities. Although the effects depend on local electricity mixes and logistics (transport, nutrient supplementation), which must be optimized in siting decisions like those for Addis Ababa, recent life‐cycle assessments demonstrate that SCP systems can achieve significantly lower climate footprints when waste‐derived substrates are used and when energy‐intensive hotspots (aeration, sterilization/drying) are powered by renewables or integrated with biogas/anaerobic digestion (Li et al. 2024; Fernández‐López et al. 2024). According to regulatory and market evaluations, reducing landfill organics reduces powerful short‐lived climate pollutants, making food waste diversion one of the most immediate levers for methane mitigation at the system level (Ahmed et al. 2023). In order to maximize resource efficiency and get closer to zero‐waste results, reviews focusing on food/fruit‐waste valorization place SCP bio‐refineries within a circular‐bio‐economy pathway that couples upstream preprocessing (sorting, pressing, and hydrolysis) with downstream microbial conversion to protein and co‐products (Taheri and Hosseini 2025). Decentralized SCP nodes close to produce markets can lower transportation emissions, recover nutrients, and produce local feed/food ingredients in Global South contexts where organic fractions predominate in municipal waste streams and methane leakage is severe, as long as biosafety and quality controls are followed (Abubakar et al. 2022).

## 8 Opportunities for Future Research and Development

### 8.1 Technological Advancements

To optimize single‐cell protein (SCP) yields from fruit wastes, focused research on effective fermentation techniques and the use of genetically modified bacteria is desperately needed: According to recent reviews, inadequate pretreatment, mass transfer in bioreactors, and incomplete substrate utilization by wild‐type strains limit conversion efficiency even though fruit by‐products are high in sugars and nutrients. These factors also limit biomass and protein productivity (Sekoai et al. 2024; Li et al. 2024). Although they require local optimization for the heterogeneous fruit substrates typical of Addis Ababa's supply chain, process engineering advancements such as fed‐batch and continuous reactors, enhanced aeration/oxygen‐transfer designs, and integrated hydrolysis–fermentation approaches have demonstrated a great deal of potential to increase volumetric productivity (Li et al. 2024; Bajić et al. 2022). Genetic and metabolic engineering advancements (CRISPR, pathway optimization, and adaptive evolution) can significantly increase carbon flux toward biomass and essential amino acid synthesis, enhance tolerance to inhibitors found in peels and pulps, and facilitate the utilization of complex carbohydrates. However, these tools have not been widely used for SCP strains used with fruit wastes and require context‐specific evaluation for regulatory acceptability and safety (Balagurunathan et al. 2022; Do et al. 2024). Thus, to turn Addis Ababa's plentiful fruit residues into a dependable, high‐yield SCP supply that is both financially feasible and nutritionally suitable for feed and food applications, targeted research that integrates strain engineering with bioprocess optimization, techno‐economic analysis, and pilot‐scale demonstrations is crucial (Li et al. 2024; Sekoai et al. 2024).

### 8.2 Policy and Regulatory Support

In order to scale sustainable waste‐to‐protein efforts, governments and regulatory agencies must establish the institutional, financial, and legal frameworks necessary to transform scattered fruit waste into safe, marketable single‐cell protein (SCP) and dependable feedstock. This entails setting clear safety and quality standards (such as feedstock specifications, microbial limits, and traceability) that reduce investment risk and safeguard public health, investing in source‐segregation and collection infrastructure to ensure processors receive consistent, contamination‐controlled biomass, and incorporating food waste valorization into solid‐waste and circular economy strategies at the municipal and national levels (Gebrekidan et al. 2024). While R&D grants and pilot‐scale facilities aid in adapting SCP technologies to local substrates and climates, regulatory tools such as targeted subsidies, tax incentives, or feedstock‐access guarantee for circular‐protein products can spur private and social‐enterprise projects (Sekoai et al. 2024). Reduced time‐to‐market for new bioprocessing plants through expedited permitting procedures and technical guidance, as well as assistance for staff training and extension services to ensure successful participation from smallholders, markets, and processors, is equally crucial. According to Teshome (2021), integrating city waste management plans with national circular‐bio‐economy aims would lessen the strain on landfills and provide feedstock for SCP pathways in Addis Ababa, where food waste predominates in municipal streams and current collection and disposal systems are inadequate. Lastly, Ethiopian regulators might modify established policy models and financing sources to suit regional goals and capacity limitations by utilizing multilateral guidelines and voluntary codes from organizations like the FAO, international technical assistance, and donor programs. To turn Addis Ababa's fruit waste into safe, dependable single‐cell protein value chains, a combination of coordinated policy measures, including regulatory standards, infrastructure investment, fiscal incentives, expedited permitting, and focused research and development partnerships, is essential (Buchner et al. 2024).

Short‐term financing, assured markets, technical assistance, and regulatory levers should all be included in potential incentives to get nearby farmers and waste‐management companies to supply fruit waste for (SCP) production: Examples include reduced permit fees or tax breaks for businesses that divert organic waste to SCP facilities, small grants or low‐interest loans to purchase collection/processing equipment (such as shredders or cold boxes), guaranteed off‐take agreements or price premiums for separated fruit residues, payments or in‐kind credits (such as compost or bio‐fertilizer) back to farmers to close nutrient loops, extension training, and basic quality/certification schemes to ensure feedstock meets fermentation needs. These initiatives are in line with Ethiopia's developing bio‐economy and circular‐economy policies, which place a high priority on the valorization of organic residues (Burrowes 2017; Gebrekidan et al. 2024). They can be implemented through public‐private partnership (PPP) frameworks, results‐based financing, and municipal incentive programs that have worked well elsewhere.

### 8.3 Collaboration With Academia and Industry

Cross‐sectoral cooperation is encouraged in Addis Ababa to lead a circular economy centered on fruit biomass by utilizing partnerships between commercial companies, NGOs, and academics. A Circular Economy Excellence Center was recently established with the specific goal of acting as “a hub for research, innovation, and capacity‐building in circular waste‐management practices,” specifically requesting platforms where stakeholders from various sectors can collaborate to develop and execute resource‐efficient solutions (Louise Bjerkli 2013; Koech et al. 2023). Addis Ababa University (Institute of Technology), the city's Environmental Protection Authority, recycling companies, and civil society already collaborate on parallel pilots under the Partnership for Circular Value Chains in Addis Ababa to operationalize circular value chains in the solid‐waste industry (Getachew 2023). Furthermore, programs like Techno‐Serve's LIWAY program, which it runs in collaboration with local partners, show how NGOs can spur micro‐enterprises that incorporate informal collectors into official recycling markets, eliminating thousands of tons of waste and creating sources of income (Nijman‐Ross et al. 2023). These examples create an emerging ecosystem where private companies can pilot and scale bioprocessing technologies, NGOs can mobilize communities and market links, and academia can provide the technical and analytical foundation for valorizing fruit waste (e.g., for single‐cell‐protein feedstocks).

## 9 Conclusion

Addis Ababa generates a considerable amount of fruit waste peels, seeds, pulp, the whole which, despite often being discarded, actually contains significant nutritional value. This review highlights the promising potential of utilizing these residues as feedstock for Single Cell Protein (SCP) production. Essentially, by employing yeasts, fungi, or bacteria, it's possible to convert this urban organic waste into valuable protein. Such an approach not only addresses food insecurity and malnutrition in low‐income urban populations but could also alleviate environmental burdens associated with landfill waste and inefficient resource use, reinforcing principles of a circular bio‐economy. The high moisture content and variable composition of fruit residues, along with seasonal fluctuations and contamination risks, complicate large‐scale SCP production. Robust pretreatment and feedstock management are essential, as are careful choices regarding microbial strains and vigilant safety monitoring for allergens and toxins. Furthermore, there are gaps in technical expertise, particularly in fermentation technology, process scaling, and downstream processing. Weak collaboration between academic, industrial, and governmental sectors further delays the transition of lab‐scale innovations to commercial reality. Economic concerns investment in collection, transport, and cold‐chain infrastructure must also be considered to ensure SCP's competitiveness against traditional protein sources. To move forward, an Ethiopia‐specific roadmap for SCP implementation is proposed. Initial steps should include pilot‐scale facilities in major market centers such as Atikilt Tera and Shola, where fruit waste is abundant and collection logistics are manageable. These pilots should focus on cost‐effective pretreatment, modular bioreactor systems, and integrated safety protocols to produce consistent, high‐quality SCP. The data and insights obtained would then inform techno‐economic analysis, life‐cycle assessments, and broader process optimization, enabling scalable, cost‐efficient, and environmentally responsible design. At the same time, capacity‐building efforts such as vocational training, short courses, and partnerships between universities, industry, and government are needed to bridge technical and regulatory gaps. Policy alignment and financial incentives play a critical role in accelerating SCP value chain development. Measures might include guaranteed access to feedstock, equipment subsidies, market guarantees, and streamlined permitting to lower investment risks and encourage private sector engagement. Public outreach through education campaigns, transparent labeling, and demonstration projects will also be crucial to building consumer trust and acceptance. In summary, fruit waste‐to‐SCP pathways represent a high‐impact, context‐appropriate solution for Ethiopia, with the potential to improve urban waste management, enhance food and nutritional security, foster local employment, and reduce environmental impacts. By integrating pilot projects, targeted research, capacity building, and supportive policy measures, Ethiopia can establish robust, economically viable, and scalable SCP systems that may serve as a model for sustainable urban protein production in other regions.

## Conflicts of Interest

The authors declare no conflicts of interest.

## Data Availability

This review article does not report any new data. All data supporting the findings discussed in this manuscript are available from previously published studies, which have been appropriately cited within the text.
